# Re‐Examination of the Reciprocal Relation Between Intrinsic Motivation and Competence Beliefs in Math Using the Random‐Intercept Cross‐Lagged Panel Framework

**DOI:** 10.1111/cdev.70028

**Published:** 2025-09-04

**Authors:** Patrick Paschke, Ricarda Steinmayr, Alica Mertens, Andreas B. Neubauer, Birgit Spinath

**Affiliations:** ^1^ Institute of Psychology Technical University Dortmund Dortmund Germany; ^2^ Institute of Psychology Heidelberg University Heidelberg Germany; ^3^ Institute of Psychology RWTH Aachen University Germany

**Keywords:** competence beliefs, intrinsic motivation, random‐intercept cross‐lagged panel model

## Abstract

Previous studies employing cross‐lagged panel models (CLPMs) found mixed results regarding reciprocal relations between competence beliefs and intrinsic motivation. CLPMs have been criticized for leading to erroneous causal inference in certain situations. Two datasets were analyzed regarding reciprocal relations between competence beliefs and intrinsic motivation with the alternative random‐intercept cross‐laged panel models (RI‐CLPMs). One dataset contained 670 2nd to 3rd grade students (*M* = 8.80 years old, 98% white, 55.7% female) and the second dataset contained 542 2nd to 4th grade students (*M* = 7.96 years old, 76.7% native German speakers, no information on ethnicity, 50.2% female) in Germany. The results revealed little to no evidence for reciprocal relations between the constructs.

Different motivational theories postulate influences of competence beliefs on intrinsic motivation (e.g., Eccles and Wigfield 2023; Harter 1981). However, longitudinal studies present mixed results. Whereas some found evidence in favor of an influence of competence beliefs on change in intrinsic motivation (e.g., Arens et al. 2019; Lauermann et al. 2017), others found no or only little evidence with small effects (e.g., Spinath and Steinmayr 2008; Vinni‐Laakso et al. 2019). A possible explanation underlying the diverging results might be the method applied by the authors. One of the most commonly used methods in this research context are cross‐lagged panel models (CLPMs). These models have recently been criticized for leading to “erroneous conclusions regarding the presence, predominance, and sign of causal influences” (Hamaker et al. 2015, 102). The proposed alternative Random‐Intercept Cross‐Lagged Panel models (RI‐CLPMs) are presented as an alternative that allows to disentangle interindividual and intraindividual changes when testing reciprocal associations between constructs. By controlling for interindividual differences, stable between‐person differences that might confound the lagged associations between constructs are removed. Thus, RI‐CLPMs account for possible person‐level confounders of the lagged associations between competence beliefs and intrinsic motivation.

In the present study, the aim was to reinvestigate the temporal relations between competence beliefs and intrinsic motivation by implementing both RI‐CLPMs (Hamaker et al. 2015) as well as traditional CLPMs. We then compare the results to facilitate a broader understanding of these temporal relations. We are using a dataset that was previously analyzed with CLPMs only (Spinath and Steinmayr 2008). Second, we use another longitudinal dataset (Weidinger et al. 2018), which has not yet been analyzed with regard to temporal relations between competence beliefs and intrinsic motivation, in order to replicate findings from analyses of the first dataset. In both datasets, we focus on the domain math because mathematical intrinsic motivation, assessed in elementary school, represents an important predictor of choices for or against STEM (Science, Technology, Engineering, and Mathematics) education and careers (e.g., Lauermann et al. 2017; Musu‐Gillette et al. 2015). Given the dearth of skilled employees in STEM (Caprile et al. 2015; Kogan and Schabinger 2023), investigating possible causal determinants of intrinsic motivation is thus of high practical relevance. In the following, we first discuss competence beliefs, intrinsic motivation, and their interconnection before turning to the utility of CLPMs and RI‐CLPMs in this particular research context.

## 1 Relations Between Competence Beliefs and Intrinsic Motivation

Competence beliefs in a certain academic domain are generally considered to be an important motivational characteristic affecting, among other factors, students' academic achievement and academic choices (e.g., Arens and Niepel 2023; Eccles and Wigfield 2020, 2023; Marsh 2022; Wigfield and Eccles 2020; Wu et al. 2021). In situated expectancy‐value theory (Eccles and Wigfield 2023; Wigfield and Eccles 2020), competence beliefs are typically operationalized as domain‐specific ability self‐concept items (e.g., Harter 1982; Herbert and Stipek 2005; Marsh 1990; Wigfield et al. 2015; Weidinger et al. 2018) that refer to the cognitive representations of one's own abilities in the respective domain (e.g., “I am good at math”; Spinath and Steinmayr 2012, 1148). Thus, in the present study we also use the term “competence beliefs” to describe the underlying construct measured by the self‐concept scales in the two datasets.

Intrinsic motivation is another prominent motivational construct that is considered important for learning in school and beyond in different motivational theories (Ryan and Deci 2019, 2020; Wigfield and Eccles 2020). As per Ryan and Deci's (2000) work (pp. 56), intrinsic motivation is characterized by engaging in an activity for its inherent satisfaction rather than for any distinct outcome.

Competence beliefs and intrinsic motivation show medium to high correlations, typically above *r* = 0.30, when assessed concurrently (e.g., *r* = 0.43 to *r* = 0.58; [Arens et al. 2019]; *r* = 0.48 to *r* = 0.59 [Niemivirta et al. 2023]; *r* = 0.29 to *r* = 0.83 [Spinath and Steinmayr 2008]; *r* = 0.36 to *r* = 0.51 [Steinmayr et al. 2018]; *r* = 0.73 [Sáinz and Upadyaya 2023]; *r* = 0.67 to *r* = 0.70 [Vinni‐Laakso et al. 2019]). In early elementary school, students typically show high competence beliefs and high intrinsic motivation for school, which then both decline during individual development (Gaspard et al. 2020; Gottfried et al. 2001; Niemivirta et al. 2023; Spinath and Steinmayr 2008; Weidinger et al. 2015, 2017). The decline in self‐concept and in intrinsic motivation often follows similar trajectories (Niemivirta et al. 2023; Weidinger et al. 2015). A recent study (Niemivirta et al. 2023) reported high correlations between initial levels (*r* = 0.90) and slopes (*r* = 0.79) for self‐concept and intrinsic motivation across time (first to third Grade). Thus, as early as in first grade, students with high self‐concepts also exhibited high intrinsic motivation, and both constructs likewise exhibited markedly similar trajectories throughout the first three grades.

Factors that are theorized to be responsible for the average decline in competence beliefs and intrinsic motivation, especially in elementary school, include performance feedback such as grades and verbal and other feedback from teachers, peers, and parents (Weidinger et al. 2015). Notably, most younger students believe themselves to be above average in math competence, as was also the case in the studies by Spinath and Steinmayr (2008) and Weidinger et al. (2018). Since grades facilitate social comparisons (Stipek and Mac Iver 1989) many of the students who believe themselves to be above average will learn over time that this is not true. Additionally, students tend to engage more in upward comparisons than in downward comparisons, which negatively affects their competence beliefs (Dickhäuser and Galfe 2004). Likewise, intrinsic motivation may suffer as a consequence of learning that one does not perform on a level as high as initially expected. Furthermore, the increasing complexity of the content in math classes might also negatively impact students' competence beliefs and intrinsic motivation. If students find math classes increasingly difficult, their math self‐concepts and intrinsic motivation might decrease even in situations where no social comparisons take place. Moreover, according to stage–environment–fit theory (Eccles et al. 1993), with increasing age, there is a greater mismatch between students' need for autonomy and the autonomy they are granted in the school system. This mismatch might negatively affect students' intrinsic motivation and possibly their competence beliefs if they reduce their learning efforts. Lastly, students' higher cognitive capacity with increasing age allows for a more realistic (and thus usually less positive) assessment of own competencies (Niemivirta et al. 2023; Wigfield and Eccles 2000). Thus, children's competence beliefs and intrinsic motivation are thought to become more negative over time due to various changes in the environment as well as developmental changes (see Wigfield and Eccles 2000). However, it is also possible that the common decline of competence beliefs and intrinsic motivation is at least partially explained by causal relations between the two constructs. Indeed, different theoretical rationales predict that competence beliefs affect subsequent intrinsic motivation: Within situated expectancy‐value theory, achievement‐related choices and performance are thought to be influenced by two major factors: A person's expectancy of success at a certain task and their subjective task values. Subjective task values consist of four components: intrinsic task value, utility task value, attainment task value, and costs. Of these four components, intrinsic task value is very closely related to intrinsic motivation (Wigfield and Eccles 2020). Intrinsic task values represent the enjoyment and gratification a person experiences when performing a certain task or type of tasks for its own sake without external reinforcement (Wigfield and Eccles 2020). Additionally, self‐concepts of own abilities are theorized to influence both expectancy of success as well as subjective task values. Thus, according to expectancy‐value theory, competence beliefs influence intrinsic motivation. The postulated effect of competence beliefs on intrinsic motivation is thought to be the result of two processes: First, students who master certain types of tasks successfully feel more competent and consequently seek similar tasks in the future. Second, students lower the values they assign to tasks for which they experience difficulties in order to preserve their global self‐worth (Eccles and Wigfield 2002).

Additionally, according to the effectance motivation model by Harter (1981), perceiving oneself as competent due to successful completion of a task is thought to lead to intrinsic pleasure associated with the task. This assumption is shared by self‐determination theory (Ryan and Deci 2019, 2020), according to which the fulfillment of the three basic psychological needs for feelings of competence, autonomy, and social relatedness facilitates intrinsic motivation. Specifically, because humans have the inherent need for feeling competent, they enjoy tasks that allow them to experience feelings of competence and thus fulfill this need (Ryan and Deci 2020).

Thus, different theories suggest that competence beliefs affect intrinsic motivation. However, it is also possible that intrinsic motivation affects competence beliefs. Regarding self‐determination theory, it has been shown that higher autonomous study motivation predicts higher subsequent need satisfaction, both on a within‐ and a between‐person level (Sosin et al. 2024). Thus, the relation between competence beliefs and intrinsic motivation might be reciprocal. Additionally, experiencing low competence feedback at a task with a high subjective task value might threaten a person's self‐worth. According to self‐worth theory (Covington 1992) protecting one's sense of academic competence is important for maintaining positive global self‐worth. Thus, students might protect their self‐worth by decreasing the subjective values of tasks they perceive as too difficult. Furthermore, students might judge their competences more optimistically if they find them intrinsically enjoyable. This could be explained in different ways, for example, by self‐serving biases, self‐worth protection, or mood effects. While there is no direct effect of intrinsic motivation on competence beliefs postulated in expectancy‐value theory, there are indirect effects. Specifically, intrinsic motivation is thought to affect achievement‐related choices and performances. The performance is then interpreted by the child with regard to its locus of control as well as causal attributions and affects competence beliefs accordingly (Eccles and Wigfield 2023). Thus, intrinsic motivation might also affect competence beliefs. However, this possibility has so far received much less attention and discussion in the literature compared with the effect of competence beliefs on intrinsic motivation.

Empirical results on the causal relations between competence beliefs and intrinsic motivation have been mixed. While some studies found effects of competence beliefs on intrinsic motivation (Arens et al. 2019; Jacobs et al. 2002; Lauermann et al. 2017), others did not (Nurmi and Aunola 2005; Skaalvik and Valås 1999; Spinath and Steinmayr 2008; Vinni‐Laakso et al. 2019), or in one case, the effect was only prevalent for male students (Sáinz and Upadyaya 2023). Those studies that revealed significant effects typically found only small effects and often only for some of the analyzed domains and waves of data. However, there is a tendency for studies with samples of secondary school students to find significant effects (Arens et al. 2019; Lauermann et al. 2017; but see Sáinz and Upadyaya 2023), while studies with samples of elementary school students typically did not (Nurmi and Aunola 2005; Spinath and Steinmayr 2008; Vinni‐Laakso et al. 2019). The studies by Jacobs et al. (2002) and Skaalvik and Valås (1999) included both elementary and secondary school students. Significant effects of intrinsic motivation on competence beliefs have not been observed or only for small subsets of the analyzed data (e.g., Arens et al. 2019; Lauermann et al. 2017; Sáinz and Upadyaya 2023; Spinath and Steinmayr 2008, 2012).

This raises the question why effects of competence beliefs on intrinsic motivation are not found consistently, even though they are incorporated in different well‐established theories. Based on Harter's (1981) theory of effectance motivation, Spinath and Steinmayr (2008) argue that it might not be the absolute level of normative competence beliefs that is important for feelings of efficacy and thus intrinsic motivation, but rather the perception of oneself as competent at successfully completing tasks relative to one's own perceived competence level. Thus, an optimal fit between task difficulty and one's own perceived competence would be more important than competence beliefs for intrinsic motivation. For example, students with high competence beliefs might not experience feelings of efficacy and thus intrinsic motivation when completing a task far below their perceived ability level, while successfully completing the same task might be rewarding for students with lower competence beliefs.

### 1.1 The Utility of RI‐CLPMs and CLPMs for Analyzing Relations Between Competence Beliefs and Intrinsic Motivation

The vast majority of studies on the effects of competence beliefs on intrinsic motivation have employed CLPMs or related models which do not disentangle within‐ and between‐person differences (e.g., Arens et al. 2019; Jacobs et al. 2002; Sáinz and Upadyaya 2023; Spinath and Spinath, 2005; Spinath and Steinmayr 2008; Vinni‐Laakso et al. 2019). Thus, these models do not allow for a differentiation between interpersonal and intrapersonal relations, while RI‐CLPMs do (Berry and Willoughby 2017; Burns et al. 2020; Hamaker 2023; Hamaker et al. 2015; Marsh et al. 2022; Mund and Nestler 2019; Usami et al. 2019). The implications of this fact are currently under debate in the literature. According to critics of CLPMs, not disentangling within‐ and between‐person differences might lead to false causal inference (Hamaker 2023; Hamaker et al. 2015; Usami et al. 2019). Of course, nonexperimental data cannot give unequivocal evidence on causality. Nevertheless, cross‐lagged designs can point out potentially causal patterns and the dominance of certain directions in comparison with others. Hamaker (2023) argues that CLPMs might be adequate for descriptive and predictive purposes, but models disentangling within‐ and between‐person differences, such as RI‐CLPMs, are better suited for causal inference. Other authors argue that RI‐CLPMs are not generally superior to CLPMs in drawing causal inference, but that both models are suited to analyze different research questions regarding causal inference (Asendorpf 2021; Orth et al. 2021; Marsh et al. 2022). In the following, we briefly summarize the main differences between CLPMs and RI‐CLPMs as well as the debate in the literature and its relevance for the particular research question of the present study.

RI‐CLPMs differ from CLPMs by the inclusion of a random intercept of each variable analyzed across time (e.g., competence beliefs and intrinsic motivation). These random intercepts are the estimated latent means of the respective variables across time and are thus invariant within subjects but differ between subjects (Hamaker et al. 2015; Marsh et al. 2022). They can be thought of as the (at least for the duration of the study) stable trait of each individual (Hamaker 2023; Hamaker et al. 2015; Marsh et al. 2022; Usami et al. 2019). By controlling for these stable between‐person differences, the latent variables at each measurement point reflect only within‐person differences, that is, the extent to which the estimated latent score at each measurement point deviates from the expected score of that person (see Marsh et al. 2022; 2706–2707). Thus, interpersonal differences (differences in the random intercepts between persons) are disentangled from intrapersonal differences (differences in the latent variable scores across time within each person). Without the inclusion of random intercepts (as in a CLPM), the latent variable scores at each measurement point include both within‐ and between‐person differences (see Allison 2009; Berry and Willoughby 2017; Hamaker et al. 2015; Marsh et al. 2022; Mund and Nestler 2019; Usami et al. 2019). This can be seen as an advantage of RI‐CLPMs because relations can clearly be attributed to one source of variance (within‐ or between‐person), while this is not possible within CLPMs (Hamaker 2023; Hamaker et al. 2015).

However, it has also been argued that RI‐CLPMs should not be considered superior to CLPMs in all cases (Asendorpf 2021; Hübner et al. 2022; Lüdtke and Robitzsch 2022; Marsh et al. 2022; Orth et al. 2021). According to these authors, RI‐CLPMs and CLPMs differ in the interpretations of their parameters and thus, are suited to analyze different research questions (see Marsh et al. 2022). We illustrate this with the example of competence beliefs predicting subsequent intrinsic motivation. Researchers interested in causal relations frequently use the cross‐lagged paths from competence beliefs on intrinsic motivation as an indicator of the effect of competence beliefs on intrinsic motivation. However, the interpretation of this “effect” differs between CLPMs and RI‐CLPMs. In a CLPM the interpretation is as follows: “Subjects with higher competence beliefs at the first measurement point (*t*_1_) relative to the other subjects in the study tend to have higher intrinsic motivation at the second measurement point (*t*_2_) relative to the other subjects in the study after controlling for their intrinsic motivation at *t*_1_.” Notably, this interpretation confounds within‐ and between‐person differences as sources of the observed relation because a person having relatively high competence beliefs compared with other individuals at *t*_1_ can be due to (1) them having relatively high stable competence beliefs (between‐person difference) or due to (2) them having relatively high current competence beliefs at this specific point in time compared with their usual competence beliefs (within‐person difference). In contrast, in an RI‐CLPM, the interpretation is: “Subjects whose competence beliefs are relatively high at *t*_1_ compared to their usual trait‐like competence beliefs across all measurement points tend to have relatively high intrinsic motivation at *t*_2_ compared to their usual trait‐like intrinsic motivation across all measurement points after controlling for intrapersonal deviations in *t*_1_ intrinsic motivation.” These two interpretations would answer quite different research questions. Thus, model selection should be based on considerations on what might facilitate intrinsic motivation: Students having high current competence beliefs compared to peers (CLPM) or students having high current competence beliefs compared to their usual trait‐like competence beliefs (RI‐CLPM)? We argue that there are theoretical reasons for both kinds of effects. Students with higher competence beliefs than their peers might also perceive that their competence beliefs are higher than their peers which could facilitate intrinsic motivation, especially in a context such as school with highly salient social comparisons (e.g., Kavanagh 2020; Lohbeck and Möller 2023; Schneider and Sparfeldt 2020). Notably, even though social comparisons should become more salient with the introduction of grades and students in Germany typically receive their first grades in Grade 3, social comparisons in German elementary school have been found in lower grades as well (Schneider and Sparfeldt 2020). However, students not only make social but also temporal comparisons and at least one study found temporal comparison processes to affect intrinsic motivation, specifically (Arens and Niepel 2023). Therefore, it seems plausible that having especially high competence beliefs at a certain time point compared to one's usual trait‐like competence beliefs could positively affect intrinsic motivation. RI‐CLPMs would be more adequate compared to CLPMs to test such an effect.

Theoretical considerations of potential causal mechanisms should inform model selection (Asendorpf 2021; Marsh et al. 2022; Orth et al. 2021). However, arguments by critics of CLPMs are largely not based on considerations of specific mechanisms of trait formation such as social or temporal comparisons (although such considerations should also be taken into account, see Hamaker 2023), but rather on more general statistical, mathematical, and philosophical considerations regarding causal inference from within‐ and between‐person sources of variance (e.g., Hamaker 2023; Hamaker et al. 2015; Usami et al. 2019). Specifically, if we propose an effect of competence beliefs on intrinsic motivation, we should be able to show that changes within a person in competence beliefs result in changes within a person in intrinsic motivation. If we observe a between‐person association between competence beliefs and intrinsic motivation but not a within‐person association, this would indicate that another mechanism such as an unobserved covariate might be responsible for the between‐person association rather than an actual causal relation between the two constructs. CLPMs cannot be used to test this because within‐person change is confounded with stable between‐person differences.

Because of this limitation, Hamaker (2023) concludes that “we need to make very strong additional assumptions, similar to those we need to make when using cross‐sectional data for causal inference” (p. 13), when drawing causal inference from CLPMs. One of these additional assumptions is the absence of the aforementioned unobserved covariates. Because the cross‐lagged paths in RI‐CLPMs reflect relations between intrapersonal sources of variance, they are more robust against the omission of time‐invariant covariates (Hamaker 2023; Hamaker et al. 2015; Marsh et al. 2022; Usami et al. 2019). For example, if gender affects both competence beliefs and intrinsic motivation and is omitted in a CLPM, spurious causal relations might be inferred because the observed cross‐lagged paths might be a result of an effect of gender on both constructs instead of a true effect of competence beliefs on intrinsic motivation. In an RI‐CLPM, this is much less of a concern as long as the effect of gender is time‐invariant (i.e., as long as gender affects competence beliefs equally at each measurement point and affects intrinsic motivation equally at each measurement point) because gender will influence the random intercepts rather than the cross‐lagged paths. This is the case because if the effect of gender is time‐invariant, it affects the stable trait‐like portion of competence beliefs and intrinsic motivation (the latent means and thus the random intercepts) rather than the time‐varying state‐like intrapersonal portion. As motivational variables such as competence beliefs and intrinsic motivation are thought to be influenced by a complex network of environmental and personal factors (e.g., Eccles and Wigfield 2023; Eccles and Wigfield 2002; Ryan and Deci 2019, 2020), it seems likely that not all covariates of competence beliefs and intrinsic motivation can be assessed in a single study. For example, according to the situated expectancy‐value model (Eccles and Wigfield 2020, 2023), children's affective reactions influence competence beliefs as well as intrinsic motivation. Thus, RI‐CLPMs can help mitigate the problem that not all theoretically relevant covariates can be included in any model. However, CLPMs can also be made more robust against time‐unvarying covariates by including lag‐2 paths (i.e., paths between nonadjacent measurement points; Marsh et al. 2022) and both types of models offer little to no protection against the omission of time‐varying covariates (see Marsh et al. 2022; Usami et al. 2019).

Studies show that conclusions can indeed differ depending on the use of CLPMs or RI‐CLPMs. For example, a study analyzing reciprocal relations between early achievement in mathematics and reading found relatively stronger associations between math performance and subsequent reading performance than between reading performance and subsequent math performance in a CLPM. In contrast, in a RI‐CLPM, no differences or even slightly larger associations between reading performance and subsequent math performance were found in the same data (Bailey et al. 2020). Similarly, Burns et al. (2020) observed reciprocal relations between self‐concepts of competence and achievement of psychology students only when applying a CLPM. Using an RI‐CLPM, they found that achievement predicted self‐concepts, but self‐concepts did not significantly predict achievement. As was to be expected since CLPMs are nested within RI‐CLPMs, in both studies, the RI‐CLPMs exhibited a better fit to the data.

In summary, the ability to test within‐person associations and the resulting relaxation of the assumption of no omitted time‐invariant covariates are, in our opinion, important advantages of RI‐CLPMs in general and for the particular research topic of the present study. Possible omitted covariates in former studies employing CLPMs could have resulted in spurious effects of competence beliefs on intrinsic motivation in some studies, but could likewise have obscured actual effects in other studies. Therefore, we consider it useful to reanalyze an existing dataset regarding this topic with RI‐CLPMs. However, it is also important to keep in mind that the interpretation of path coefficients differs between RI‐CLPMs and CLPMs, as has been discussed. Thus, both model types can offer insights into temporal relations that the other cannot.

## 2 The Present Study

In the present study, we analyze two datasets (Spinath and Steinmayr 2008; Weidinger et al. 2018) with RI‐CLPMs and traditional CLPMs. We also contrast cross‐lagged models with simpler autoregressive models in which the cross‐lagged paths are fixed to zero. Thus, four types of models are contrasted: cross‐lagged RI‐CLPMs, with vs. without cross‐regressive paths, and cross‐lagged CLPMs, with vs. without cross‐regressive paths. We then compare the results of these four model types in each dataset with regard to their model fits and path estimates (concurrent paths between the constructs, autoregressive paths, and most importantly, cross‐lagged paths). Thus, we aim to answer the following research questions (RQs) which are, with the exception of RQ1, based on those issued by Spinath and Steinmayr (2008):

1. Which model type is best suited to describe concurrent and time‐shifted relations between competence beliefs and intrinsic motivation: Cross‐lagged RI‐CLPMs, autoregressive RI‐CLPMs, cross‐lagged CLPMs, or autoregressive CLPMs?
2. Are there concurrent associations between competence beliefs and intrinsic motivation?
3. Can competence beliefs and/or intrinsic motivation be predicted from the same construct at prior time points?
4. Can competence beliefs and/or intrinsic motivation be predicted by the respective other construct at prior time points?

## 3 Method

### 3.1 Dataset 1 (Spinath and Steinmayr 2008)

#### 3.1.1 Participants

In 2002 and 2003, 664 students from 29 classes in 13 German elementary schools in North‐Rhine Westphalia took part in the study. The students were surveyed at four measurement occasions with a time interval of 3 to 4 months between each measurement point (see Table 1 in the results section for details). The children attended second grade (June 2002) at the first measurement point and third grade (May 2003) at the fourth measurement point. A number of *n* = 297 children were male (44.7%); *n* = 344 children were female (51.8%); and *n* = 23 (3.5%) did not report their gender. Additionally, 98% reported being white (no further specification for the remaining 2% was assessed) with German citizenship (86%). Of the remaining 14%, the majority (8% of the entire sample) had Turkish citizenship and 6% had the citizenship of another not specified country. At the first measurement occasion, children were on average *M* = 8.81 (SD = 0.56) years old. For further details on the sample, please refer to the original study report (Spinath and Steinmayr 2008).

**TABLE 1:** Means and standard deviations of competence beliefs and intrinsic motivation in both datasets.

Dataset 1 (Spinath and Steinmayr 2008)	Competence beliefs				Intrinsic motivation			
	*M*	SD	Skewness	Kurtosis	*M*	SD	Skewness	Kurtosis
June 2002 (*t*_S1_)	4.05	0.89	−0.79	0.08	3.89	1.00	−0.62	−0.52
October 2002 (*t*_S2_)	3.98	0.88	−0.67	−0.21	3.76	0.99	−0.61	−0.35
January 2003 (*t*_S3_)	3.96	0.96	−0.80	0.04	3.79	1.01	−0.70	−0.22
May 2003 (*t*_S4_)	3.98	0.88	−0.70	0.22	3.81	1.05	−0.78	−0.09

Dataset 2 (Weidinger et al. 2018)	*M*	SD	Skewness	Kurtosis	*M*	SD	Skewness	Kurtosis
July 2009 (*t*_W1_)	4.00	0.82	−0.70	0.15	3.95	1.11	−0.99	0.21
November 2009 (*t*_W2_)	3.94	0.78	−0.48	−0.19	4.00	0.97	−0.95	0.47
March 2010 (*t*_W3_)	3.87	0.78	−0.48	0.45	3.97	0.99	−0.94	0.42
July 2010 (*t*_W4_)	3.80	0.82	−0.44	0.13	3.80	1.07	−0.66	−0.36
November 2010 (*t*_W5_)	3.92	0.76	−0.46	0.11	3.87	1.03	−0.76	−0.06
March 2011 (*t*_W6_)	3.84	0.78	−0.29	−0.11	3.83	0.95	−0.70	0.09
July 2001 (*t*_W7_)	3.78	0.74	−0.17	−0.06	3.69	1.03	−0.57	−0.28

#### 3.1.2 Design and Procedure

In the original study, the students' parents provided written informed consent and agreed to their child's participation in the study. The complete testing took about 30 min, also including other measures that were not part of the present work (please refer to Spinath and Steinmayr (2008) for further details regarding the respective measures). Besides demographic measures (e.g., age and gender), intrinsic motivation and competence beliefs with respect to math and two other domains (German and school in general) were assessed. Teachers who did not teach in the tested class administered the test sessions.

#### 3.1.3 Measures

##### 3.1.3.1 Intrinsic Values

Intrinsic motivation regarding math was assessed using three items. The children indicated on a 5‐point Likert‐type scale ranging from *very much* (1) to *not at all* (5) how much they liked three respective activities (“How much do you like mental arithmetic/math problems/doing calculations?”). To achieve a more intuitive understanding of the respective scale, we recoded the respective items. Thus, higher scores indicate higher intrinsic values. Internal consistency (McDonald's omega) ranged from *ω* = 0.99 to *ω* = 1.00 across the measurement points. The test–retest reliabilities between the adjacent measurement points ranged from *r*_tt_ = 0.66 to *r*_tt_ = 0.85 (see Spinath and Steinmayr 2008 for further details).

##### 3.1.3.2 Competence Beliefs

Math competence beliefs were also measured by means of three items. Children could answer on a 5‐point Likert‐type scale. The following questions were used: “How good are you at math?” with response choice ranging from *very good* (1) to *very bad* (5); “How easy is it for you to learn new things in math?” with response option from *very easy* (1) to *very hard* (5); and “To which group of students do you belong in math?” ranging from *the best* (1) to *the worst* (5). Again, the respective items were recoded so that the scale could be interpreted more intuitively with higher values indicating higher competence beliefs. Internal consistency was *ω* = 0.99 at all measurement points and retest reliabilities ranged from *r*_tt_ = 0.45 to *r*_tt_ = 0.87.

#### 3.1.4 Statistical Analysis

**FIGURE 1:**
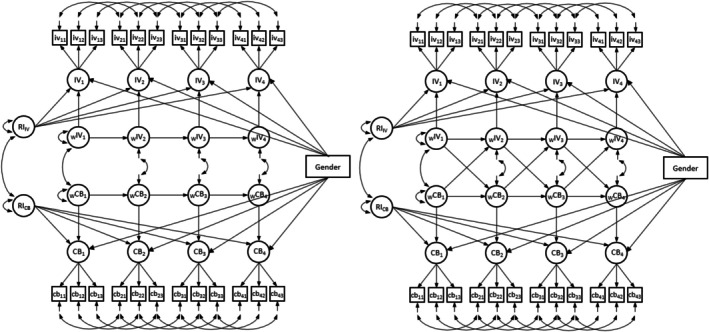
Visualization of the autoregressive (left) and cross‐lagged model (right) using the random‐intercept cross‐lagged panel framework.

We used analyses of variance (ANOVA) with repeated measures in IBM SPSS Statistics 29 to test whether intrinsic motivation and competence beliefs differed significantly between time points. We used the Mplus version 8.501 (Muthén and Muthén 2017) to employ the structural equation models. Two types of models were constructed: Multi‐Indicator RI‐CLPMs (see Mulder and Hamaker 2021) and traditional CLPMs. Three items indicated each latent variable (competence beliefs and intrinsic motivation) in both model types (RI‐CLPMs and traditional CLPMs). We used the raw scores (i.e., not transformed scores) of the items as indicators. For each model type, we set up two competing models, one including the reciprocal effects (cross‐lagged model) and one without the cross‐lagged effects (autoregressive model). For a visualization, see Figure 1. Moreover, the residual covariances between the same items at different measurement points were estimated freely. We also incorporated gender (coded as 1 = male, 2 = female) as a predictor of competence beliefs and intrinsic motivation at each measurement point. This allowed us to control for potential time‐variant effects of gender (e.g., the effect of gender becoming more pronounced with age; see Mulder and Hamaker 2021, 14–15). We also controlled for the nested data structure (students nested in classes) by the use of Mplus' type = complex function and class as the cluster variable.

Because the autoregressive models are nested within the cross‐lagged models and the CLPMs are also nested within the RI‐CLPMs, we compared the models by the Akaike information criterion (AIC; Akaike 1973; Kuha 2004) and the Bayesian information criterion (BIC; Kuha 2004; Schwarz 1978). Additionally, we report the following criteria: the *χ* ^2^‐value, the RMSEA, the CFI, and the SRMR, using the criteria laid out by Hu and Bentler (1999) for adequate model fit (CFI > 0.95, SRMR < 0.08, and RMSEA < 0.06). The MLR estimator in Mplus was used for the estimation of robust standard errors.

##### 3.1.4.1 Missing Values

As not all students participated at all four measurement occasions, we had to account for missing values in the data. In the study by Spinath and Steinmayr (2008), no systematic difference was found in any investigated variable between those students who provided complete datasets and those who did not. Moreover, the amount of missing data for individual items was small (less than 1%). Thus, we decided to use the full‐information maximum likelihood (FIML) estimator in order to maximize statistical power by including as many cases as possible.

##### 3.1.4.2 Measurement Invariance

We tested intrinsic motivation and competence beliefs for four levels of measurement invariance across the four measurement time points: configural invariance (equal factor structure), metric invariance (additionally, equal factor loadings), scalar invariance (additionally, equal item intercepts), and residual invariance (additionally, equal residual variances). We compared the four invariance models by differences in the RMSEA, CFI, and SRMR. We used differences in the CFI of 0.01, differences in the RMSEA of 0.015, and differences in SRMR of 0.03 for metric invariance, as well as 0.015 for scalar and residual invariance to evaluate measurement invariance (Chen 2007). Both intrinsic values and competence beliefs demonstrated residual invariance (see Data S1 (OSM 1) for details) and thus, the respective assumptions were included in the models.

### 3.2 Dataset 2 (Weidinger et al. 2018)

#### 3.2.1 Participants

The second dataset that we used to implement the RI‐CLPM stems from a longitudinal project that aimed to investigate the development of motivation during elementary school (MEGA, MotivationsEntwicklung im GrundschulAlter, see Weidinger et al. 2015, 2017, 2018, 2019). Five hundred forty‐two students from second grade took part in the study. The gender distribution was nearly even (*n* = 272 (50.2%) female, *n* = 270 (49.8%) male). Additionally, *n* = 293 students (54.1%) reported German as their first language; *n* = 89 students (16.4%) chose “other language”; and *n* = 160 students (29.5%) did not report their first language. The students were from 27 classes nested in 11 schools. Data were collected at seven measurement occasions (between July 2009 and July 2011) with a time interval of 4 months between the measurement points. At the first measurement occasion, children were on average *M* = 7.96 (SD = 0.58) years old. For further details regarding the respective sample, please refer to the original study report (Weidinger et al. 2018).

#### 3.2.2 Design and Procedure

About 75% of all parents provided signed informed consent in the original study so that about 75% of the basic student population was tested. All variables were assessed during regular class sessions in the students' classrooms. Trained research assistants performed the testing. All items were read aloud to the children to ensure the same working speed. In total, the testing took about 45 min. Again, demographic measures (e.g., age and gender), intrinsic motivation, and competence beliefs with respect to math (among other measures, see Weidinger et al. 2018) were assessed.

#### 3.2.3 Measures

##### 3.2.3.1 Intrinsic Values

Intrinsic motivation regarding math was measured with the established scale that was used in the publication by Spinath and Steinmayr (2008). On a 5‐point Likert‐type scale ranging from *very much* (1) to *not at all* (5) the children indicated how much they liked three respective activities. The items were recoded so that higher values referred to higher intrinsic motivation. Internal consistency (McDonald's omega) ranged from *ω* = 0.78 to *ω* = 0.90.

##### 3.2.3.2 Competence Beliefs

To assess math competence beliefs, the same measure like in the investigation of Spinath and Steinmayr (2008) was used. Again, the students responded on a 5‐point Likert‐type scale ranging from 1 (*very good*/*very easy*/*the best*) to 5 (*very bad*/*very hard*/*the worst*) to the three items. Again, the items were recoded to reach a more intuitive understanding of the scale. Internal consistency ranged from *ω* = 0.80 to *ω* = 0.90.

#### 3.2.4 Statistical Analysis

The statistical approach to analyze the data from the MEGA project was comparable to the analyses used for Dataset 1. Instead of four measurement occasions, we modeled seven measurement points. Each latent variable was again indicated by three items. In Dataset 2, controlling for the nested data structure was not possible since it led to nonconvergence of the models. We empirically estimated the proportion of variance on the scale level (intrinsic motivation, competence beliefs) that resulted from the clustered data structure (students in classes) relative to the total variance. We did so by computing intraclass correlations (ICCs) in three‐level models with repeated measurements (level 1: time; level 2: students; level 3: classes) and random intercepts on levels two and three. The results revealed that only small proportions of variance were observed on level 3 (Dataset 1: intrinsic motivation: 3.1%; competence beliefs: 2.1%; Dataset 2: intrinsic motivation: 1.3%; competence beliefs: 0.1%). Additionally to the mentioned analyses, we tested a model with larger time intervals (using the first, fourth, and seventh measurement point) in order to investigate whether reciprocal effects occur with respect to longer time periods.

##### 3.2.4.1 Missing Data

As in Dataset 1, there was little missing data due to nonresponse. For competence beliefs, between 0.0% (*t*_2_ and *t*_3_) and 0.8% (*t*_4_) were missing due to nonresponse. For intrinsic motivation, these numbers ranged from 0.0% (*t*_6_ and *t*_7_) to 0.2% (*t*_1_ and *t*_3_). Between 13.5% (*t*_4_) and 17.3% (*t*_7_) of students were missing entirely on the testing days, according to Weidinger et al. (2018) mostly due to illnesses. The FIML estimator was used. Please refer to Weidinger et al. (2018) for further details regarding the missing values.

##### 3.2.4.2 Measurement Invariance

We tested the same measurement invariance models as in Dataset 1. Competence beliefs demonstrated scalar invariance and intrinsic values demonstrated metric invariance (see OSM 1). The respective assumptions were included in the models. Analyses were confirmatory in the sense that hypotheses were derived from theoretical considerations and tested. Empirical results of the present study did not affect methodology in any way.

## 4 Results

### 4.1 Preliminary Results

Means, standard deviations, skewness, and kurtosis of competence beliefs and intrinsic motivation are reported in Table 1. Histograms of the competence belief scales and intrinsic motivation scales are reported in OSM 2. Mauchly tests indicated that the assumption of sphericity was violated in the ANOVAs (Dataset 1 competence beliefs: *χ* ^2^(5) = 50.20, *p* < 0.001; Dataset 1 intrinsic motivation: *χ* ^2^(5) = 58.57, *p* < 0.001; Dataset 2 competence beliefs: *χ* ^2^(20) = 128.54, *p* < 0.001; Dataset 2 intrinsic motivation: *χ* ^2^(20) = 193.97, *p* < 0.001). Thus, we used a Huynh–Feldt correction of the degrees of freedom (0.79 ≤ ε ≤ 0.92). The ANOVAs indicated that intrinsic motivation (*F*(2.73, 967.90) = 3.01, *p* = 0.034, *η* ^2^ = 0.01) but not competence beliefs (*F*(2.77, 954.32) = 2.58, *p* = 0.057, *η* ^2^ = 0.01) differed significantly across time in the first dataset (Spinath and Steinmayr 2008). In the second dataset (Weidinger et al. 2018), both intrinsic motivation (*F*(4.73, 1244.63) = 9.06, *p* < 0.001, *η* ^2^ = 0.03) and competence beliefs (*F*(4.98, 1305.41) = 6.12, *p* < 0.001, *η* ^2^ = 0.02) differed significantly across time. Results for all pairwise comparisons are reported in Table 2. We used a Šidák correction of the alpha level in the pairwise comparisons, resulting in an individual alpha level of *α* = 0.008 in the case of Dataset 1 and *α* = 0.002 in the case of Dataset 2. Of particular interest were the pairwise comparisons between adjacent measurement points as they indicated in which intervals the students' intrinsic motivation and/or competence beliefs changed significantly. Given the aforementioned alpha levels, there were no significant pairwise comparisons in Dataset 1 for either construct. In Dataset 2, intrinsic motivation declined significantly between *t*_3_ and *t*_4_ (Δ*M* = 0.16, *p* < 0.001) and between *t*_6_ and *t*_7_ (Δ*M* = 0.17, *p* < 0.001), while competence beliefs did not change significantly within any interval. However, there were some significant differences between nonadjacent measurement points (for details, see Table 2). While both competence beliefs and intrinsic motivation also increased between some intervals in both datasets, none of these changes were significant. Thus, while there is evidence from the main effect of time that intrinsic motivation changed over time in both datasets and competence beliefs changed over time in Dataset 2, there is relatively little evidence for change within specific intervals due to the large number of individual comparisons and resulting low individual alpha levels.

**TABLE 2:** Mean differences in intrinsic motivation and competence beliefs between the measurement points and *p* values in brackets.

Dataset 1	Intrinsic motivation			Competence beliefs		
	*t*_*S*2_	*t*_*S*3_	*t*_*S*4_	*t*_*S*2_	*t*_*S*3_	*t*_*S*4_
*t*_*S*1_	0.12 (0.013)	0.11 (0.032)	0.13 (0.019)	0.04 (0.263)	0.08 (0.068)	0.11 (0.018)
*t*_*S*2_		−0.01 (0.902)	0.01 (0.853)		0.04 (0.317)	0.06 (0.145)
*t*_*S*3_			0.02 (0.702)			0.03 (0.467)

Dataset 2	Intrinsic motivation						Competence beliefs					
	*t*_*W*2_	*t*_*W*3_	*t*_*W*4_	*t*_*W*5_	*t*_*W*6_	*t*_*W*7_	*t*_*W*2_	*t*_*W*3_	*t*_*W*4_	*t*_*W*5_	*t*_*W*6_	*t*_*W*7_
*t*_*W*1_	−0.00 (0.956)	0.08 (0.247)	0.24 (< 0.001)	0.13 (0.039)	0.13 (0.041)	0.30 (< 0.001)	0.08 (0.062)	0.13 (0.005)	0.17 (< 0.001)	0.10 (0.027)	0.13 (0.008)	0.22 (< 0.001)
*t*_*W*2_		0.08 (0.140)	0.24 (< 0.001)	0.14 (0.017)	0.13 (0.015)	0.31 (< 0.001)		0.05 (0.192)	0.09 (0.024)	0.02 (0.589)	0.05 (0.199)	0.14 (0.001)
*t*_*W*3_			0.16 (< 0.001)	0.06 (0.226)	0.06 (0.267)	0.23 (< 0.001)			0.04 (0.311)	−0.03 (0.421)	0.00 (0.950)	0.09 (0.018)
*t*_*W*4_				−0.11 (0.016)	−0.11 (0.017)	0.07 (0.158)				−0.07 (0.057)	−0.04 (0.353)	0.05 (0.113)
*t*_*W*5_					−0.00 (0.955)	0.17 (< 0.001)					0.03 (0.345)	0.12 (< 0.001)
*t*_*W*6_						0.17 (< 0.001)						0.09 (0.003)

*Note:* Dataset 1 = Spinath & Steinmayr et al. (2018); Dataset 2 = Weidinger et al. (2018).

The random intercepts correlated positively and significantly in all RI‐CLPMs (Dataset 1 cross‐lagged: *r* = 0.84, *p* < 0.001; Dataset 1 autoregressive: *r* = 0.89, *p* < 0.001; Dataset 2, seven time points cross‐lagged: *r* = 0.73, *p* < 0.001; Dataset 2, seven time points autoregressive: *r* = 0.70, *p* < 0.001; Dataset 2, three time points cross‐lagged: *r* = 0.63, *p* = 0.047; Dataset 2, three time points autoregressive: *r* = 0.77, *p* < 0.001). The model fit indices and information criteria for both datasets and model types (RI‐CLPMs and CLPMs) are reported in Table 3. We analyzed concurrent correlations between the two constructs (Table 4) because prior studies have shown competence beliefs and intrinsic motivation to be highly correlated. We also report autoregressive paths (e.g., from *t*_1_ competence beliefs on *t*_2_ competence beliefs; Table 5). Lastly, and most importantly, we calculated cross‐lagged effects (only for the cross‐lagged models) to indicate whether there were reciprocal influences between competence beliefs and intrinsic motivation on the individual level (Table 6). Note that all path coefficients reported in the Tables as well as in the main text are standardized coefficients. We first compared the respective models by their model fit and information criteria before turning to the coefficients of the individual models.

**TABLE 3:** Indicators of model fit of the autoregressive and cross‐lagged models for the two datasets using the RI‐CLPM and the CLPM. In the second dataset (Weidinger et al. 2018), the additional model incorporating measurement occasions 1, 4, and 7 is also reported.

Dataset	Paths	Model	*χ* ^2^	df	*χ* ^2^/df	RMSEA	SRMR	CFI	AIC	BIC	Best fitting model
1 (Spinath and Steinmayr 2008)	Autor.	RI‐CLPM	374.09	267	1.40	0.025	0.047	0.981	32774.96	33139.32	**Yes**
	Cross‐L.	RI‐CLPM	366.74	261	1.41	0.025	0.045	0.981	32777.04	33168.39	No
	Autor.	CLPM	401.90	270	1.49	0.027	0.057	0.976	32809.50	33160.37	No
	Cross‐L.	CLPM	384.80	264	1.46	0.026	0.050	0.978	32799.91	33177.77	No
2 (Weidinger et al. 2018; 7 time points)	Autor.	RI‐CLPM	1126.10	741	1.52	0.031	0.069	0.969	41133.94	42190.57	**Yes**
	Cross‐L.	RI‐CLPM	1098.45	729	1.51	0.031	0.071	0.970	41122.23	42230.41	**Yes**
	Autor.	CLPM	1190.51	744	1.60	0.033	0.086	0.964	41206.31	42250.06	No
	Cross‐L.	CLPM	1168.07	732	1.60	0.033	0.082	0.965	41200.19	42295.48	No
2 (Weidinger et al. 2018; 3 time points)	Autor.	RI‐CLPM	266.71	133	2.01	0.043	0.058	0.969	19182.46	19499.49	Undecid.
	Cross‐L.	RI‐CLPM	258.59	129	2.00	0.043	0.055	0.970	19181.67	19515.83	Undecid.
	Autor.	CLPM	274.78	136	2.02	0.044	0.064	0.967	19189.75	19493.92	Undecid.
	Cross‐L.	CLPM	261.87	132	1.98	0.043	0.056	0.970	19181.17	19502.48	Undecid.

Abbreviations: AIC, Akaike information criterion; Autor., autoregressive; BIC, Bayesian information criterion; CFI, Comparative Fit Index; Cross‐L., cross‐lagged; RMSEA, Root Mean Square Error of Approximation; SRMR, Standardized Root Mean Squared Residual; Undecid., undecided.

**TABLE 4:** Concurrent correlations in the RI‐CLPMs and CLPMs.

Dataset 1	RI‐CLPM						CLPM					
	Cross‐lagged model			Autoregressive model			Cross‐lagged model			Autoregressive model		
	*r*	SE	*p*	*r*	SE	*p*	*r*	SE	*p*	*r*	SE	*p*
*t*_S1_ CB ↔ IV	0.57	0.14	< 0.001	0.47	0.15	0.002	0.74	0.04	< 0.001	0.80	0.03	< 0.001
*t*_S2_ CB ↔ IV	0.77	0.12	< 0.001	0.69	0.08	< 0.001	0.68	0.06	< 0.001	0.76	0.06	< 0.001
*t*_S3_ CB ↔ IV	0.67	0.10	< 0.001	0.65	0.09	< 0.001	0.69	0.09	< 0.001	0.71	0.08	< 0.001
*t*_S4_ CB ↔ IV	0.50	0.14	< 0.001	0.49	0.13	< 0.001	0.56	0.12	< 0.001	0.57	0.12	< 0.001

Datasets 2 and 7 time points	Cross‐lagged model			Autoregressive model			Cross‐lagged model			Autoregressive model		
	*r*	SE	*p*	*r*	SE	*p*	*r*	SE	*p*	*r*	SE	*p*
*t*_W1_ CB ↔ IV	0.76	0.07	< 0.001	0.74	0.08	< 0.001	0.72	0.05	< 0.001	0.72	0.05	< 0.001
*t*_W2_ CB ↔ IV	0.70	0.12	< 0.001	0.69	0.08	< 0.001	0.65	0.07	< 0.001	0.64	0.06	< 0.001
*t*_W3_ CB ↔ IV	0.33	0.13	0.004	0.39	0.09	< 0.001	0.58	0.07	< 0.001	0.54	0.06	< 0.001
*t*_W4_ CB ↔ IV	0.51	0.11	< 0.001	0.54	0.09	< 0.001	0.61	0.08	< 0.001	0.64	0.06	< 0.001
*t*_W5_ CB ↔ IV	0.48	0.09	< 0.001	0.50	0.08	< 0.001	0.53	0.08	< 0.001	0.55	0.08	< 0.001
*t*_W6_ CB ↔ IV	0.53	0.08	< 0.001	0.52	0.07	< 0.001	0.56	0.08	< 0.001	0.58	0.07	< 0.001
*t*_W7_ CB ↔ IV	0.58	0.07	< 0.001	0.56	0.07	< 0.001	0.62	0.07	< 0.001	0.62	0.07	< 0.001

Datasets 2 and 3 time points	Cross‐lagged model			Autoregressive model			Cross‐lagged model			Autoregressive model		
	*r*	SE	*p*	*r*	SE	*p*	*r*	SE	*p*	*r*	SE	*p*
*t*_W1_ CB ↔ IV	0.74	0.10	< 0.001	0.70	0.08	< 0.001	0.71	0.05	< 0.001	0.71	0.05	< 0.001
*t*_W4_ CB ↔ IV	0.56	0.09	< 0.001	0.51	0.11	< 0.001	0.57	0.06	< 0.001	0.59	0.05	< 0.001
*t*_W7_ CB ↔ IV	0.47	0.07	< 0.001	0.46	0.06	< 0.001	0.47	0.06	< 0.001	0.51	0.06	< 0.001

**TABLE 5:** Autoregressive paths in the RI‐CLPMs and CLPMs.

Dataset 1	RI‐CLPM						CLPM					
	Cross‐lagged model			Autoregressive model			Cross‐lagged model			Autoregressive model		
	*β*	SE	*p*	*β*	SE	*p*	*β*	SE	*p*	*β*	SE	*p*
*t*_S1_–*t*_S2_ CB → CB	0.40	0.16	0.013	0.27	0.18	0.128	0.67	0.06	< 0.001	0.76	0.03	< 0.001
*t*_S2_–*t*_S3_ CB → CB	0.64	0.29	0.024	0.46	0.10	< 0.001	0.77	0.09	< 0.001	0.78	0.02	< 0.001
*t*_S3_–*t*_S4_ CB → CB	0.55	0.22	0.011	0.52	0.08	< 0.001	0.75	0.06	< 0.001	0.80	0.03	< 0.001
*t*_S1_–*t*_S2_ IV → IV	0.08	0.28	0.785	0.37	0.14	0.008	0.59	0.08	< 0.001	0.81	0.02	< 0.001
*t*_S2_–*t*_S3_ IV → IV	0.11	0.68	0.868	0.42	0.14	0.002	0.79	0.10	< 0.001	0.81	0.04	< 0.001
*t*_S3_–*t*_S4_ IV → IV	0.65	0.26	0.011	0.59	0.10	< 0.001	0.90	0.10	< 0.001	0.84	0.04	< 0.001

Datasets 2 and 7 time points	Cross‐lagged model			Autoregressive model			Cross‐lagged model			Autoregressive model		
	*β*	SE	*p*	*β*	SE	*p*	*β*	SE	*p*	*β*	SE	*p*
*t*_W1_–*t*_W2_ CB → CB	0.24	0.17	0.162	0.32	0.11	0.004	0.76	0.08	< 0.001	0.68	0.05	< 0.001
*t*_W2_–*t*_W3_ CB → CB	0.41	0.29	0.148	0.29	0.14	0.038	0.83	0.08	< 0.001	0.77	0.03	< 0.001
*t*_W3_–*t*_W4_ CB → CB	0.53	0.15	< 0.001	0.45	0.13	< 0.001	0.90	0.04	< 0.001	0.83	0.03	< 0.001
*t*_W4_–*t*_W5_ CB → CB	0.50	0.14	< 0.001	0.60	0.11	< 0.001	0.84	0.04	< 0.001	0.89	0.02	< 0.001
*t*_W5_–*t*_W6_ CB → CB	0.70	0.13	<. 001	0.64	0.10	< 0.001	0.95	0.04	< 0.001	0.88	0.02	< 0.001
*t*_W6_–*t*_W7_ CB → CB	0.73	0.08	< 0.001	0.69	0.06	< 0.001	0.93	0.03	< 0.001	0.88	0.02	< 0.001
*t*_W1_–*t*_W2_ IV → IV	0.22	0.22	0.319	0.38	0.12	0.001	0.58	0.09	< 0.001	0.72	0.04	< 0.001
*t*_W2_–*t*_W3_ IV → IV	0.46	0.24	0.060	0.22	0.17	0.185	0.81	0.08	< 0.001	0.73	0.04	< 0.001
*t*_W3_–*t*_W4_ IV → IV	0.31	0.16	0.053	0.46	0.14	0.001	0.71	0.06	< 0.001	0.80	0.03	< 0.001
*t*_W4_–*t*_W5_ IV → IV	0.70	0.11	< 0.001	0.68	0.09	< 0.001	0.87	0.04	< 0.001	0.88	0.02	< 0.001
*t*_W5_–*t*_W6_ IV → IV	0.50	0.12	< 0.001	0.58	0.10	< 0.001	0.76	0.05	< 0.001	0.83	0.03	< 0.001
*t*_W6_–*t*_W7_ IV → IV	0.69	0.09	< 0.001	0.71	0.07	< 0.001	0.84	0.04	< 0.001	0.88	0.02	< 0.001

Datasets 2 and 3 time points	Cross‐lagged model			Autoregressive model			Cross‐lagged model			Autoregressive model		
	*β*	SE	*p*	*β*	SE	*p*	*β*	SE	*p*	*β*	SE	*p*
*t*_W1_–*t*_W4_ CB → CB	0.54	0.32	0.095	0.07	0.22	0.746	0.60	0.10	< 0.001	0.50	0.06	< 0.001
*t*_W4_–*t*_W7_ CB → CB	0.76	0.13	< 0.001	0.55	0.11	< 0.001	0.81	0.06	< 0.001	0.76	0.04	< 0.001
*t*_W1_–*t*_W4_ IV → IV	−0.03	0.30	0.934	0.22	0.25	0.381	0.40	0.09	< 0.001	0.51	0.05	< 0.001
*t*_W4_–*t*_W7_ IV → IV	0.41	0.15	0.007	0.59	0.17	< 0.001	0.62	0.07	< 0.001	0.75	0.04	< 0.001

**TABLE 6:** Cross‐paths and paths from gender in the cross‐lagged RI‐CLPMs and CLPMs.

Dataset 1	RI‐CLPMs			CLPMs		
	*β*	SE	*p*	*β*	SE	*p*
*t*_S1_–*t*_S2_ CB → IV	0.41	0.19	0.035	0.26	0.09	0.005
*t*_S2_–*t*_S3_ CB → IV	0.35	0.60	0.559	0.03	0.11	0.786
*t*_S3_–*t*_S4_ CB → IV	−0.10	0.22	0.658	−0.06	0.10	0.535
*t*_S1_–*t*_S2_ IV → CB	0.04	0.15	0.779	0.12	0.07	0.080
*t*_S2_–*t*_S3_ IV → CB	−0.14	0.27	0.619	0.02	0.10	0.822
*t*_S3_–*t*_S4_ IV → CB	−0.02	0.28	0.958	0.06	0.07	0.370

Datasets 2 and 7 time points	*β*	SE	*p*	*β*	SE	*p*
*t*_W1_–*t*_W2_ CB → IV	0.28	0.22	0.221	0.18	0.09	0.049
*t*_W2_–*t*_W3_ CB → IV	−0.37	0.19	0.057	−0.12	0.09	0.157
*t*_W3_–*t*_W4_ CB → IV	0.04	0.15	0.767	0.11	0.06	0.061
*t*_W4_–*t*_W5_ CB → IV	−0.20	0.12	0.099	0.00	0.05	0.937
*t*_W5_–*t*_W6_ CB → IV	0.05	0.12	0.695	0.10	0.06	0.095
*t*_W6_–*t*_W7_ CB → IV	−0.06	0.09	0.503	0.06	0.05	0.236
*t*_W1_–*t*_W2_ IV → CB	0.10	0.16	0.549	−0.07	0.08	0.377
*t*_W2_–*t*_W3_ IV → CB	−0.19	0.29	0.527	−0.07	0.09	0.427
*t*_W3_–*t*_W4_ IV → CB	−0.35	0.11	0.001	−0.09	0.06	0.090
*t*_W4_–*t*_W5_ IV → CB	0.12	0.11	0.310	0.08	0.05	0.126
*t*_W5_–*t*_W6_ IV → CB	−0.18	0.10	0.087	−0.08	0.05	0.130
*t*_W6_–*t*_W7_ IV → CB	−0.09	0.07	0.201	−0.07	0.04	0.110

Datasets 2 and 3 time points	*β*	SE	*p*	*β*	SE	*p*
*t*_W1_–*t*_W4_ CB → IV	0.34	0.30	0.264	0.12	0.10	0.222
*t*_W4_–*t*_W7_ CB → IV	0.32	0.14	0.027	0.21	0.07	0.003
*t*_W1_–*t*_W4_ IV → CB	−0.16	0.23	0.482	−0.10	0.10	0.275
*t*_W4_–*t*_W7_ IV → CB	−0.04	0.10	0.672	−0.04	0.07	0.539

### 4.2 RQ1—Model Fit and Model Comparison

Overall, most models exhibited good model fit and met the established cutoff criteria for the fit indices (see Table 3; CFI > 0.95, SRMR < 0.08, RMSEA < 0.06; Hu & Hu and Bentler 1999). The exception was the autoregressive CLPM and the cross‐lagged CLPM in Dataset 2 with seven measurement points, which did not meet the cutoff criterion for the SRMR (autoregressive CLPM: SRMR = 0.086; cross‐lagged CLPM: SRMR = 0.082). Thus, it appeared that in Dataset 1 and Dataset 2 with three measurement points, all models achieved good model fit, while the inclusion of random intercepts as in the RI‐CLPMs was necessary for that in Dataset 2 with seven measurement points. In the following, the comparison between the models is based on the AIC and BIC. However, the CFI, RMSEA, and SRMR of the best fitting model(s) are reported for completeness.

In Dataset 1, the autoregressive RI‐CLPM (CFI = 0.981; SRMR = 0.047; RMSEA = 0.025) could be considered the best model. Both its AIC and BIC values were the lowest in this dataset with differences of at least ΔAIC = 2.08 and ΔBIC = 21.05 compared to all other models.

In Dataset 2 with seven measurement points, the two RI‐CLPMs outperformed the two CLPMs, but none of the two RI‐CLPMs was clearly superior to the other. The differences in the AIC values between RI‐CLPMs and corresponding CLPMs ranged from ΔAIC = 72.37 to ΔAIC = 77.96, and the differences in the BIC values ranged from ΔBIC = 59.49 to ΔBIC = 65.07. Between the two RI‐CLPMs, the autoregressive one exhibited the better BIC (ΔBIC = 39.84) and the cross‐lagged one exhibited the better AIC (ΔAIC = 11.71). This result can be explained by the fact that the BIC penalizes models with higher complexity more heavily than the AIC in most cases (Narisetty 2020). While we do not think that there is a particular reason to value the information of the AIC or BIC more strongly in general, we do think that a case can be made to favor the simpler model in this particular instance. Since both models exhibited highly similar and good model fit according to the aforementioned cutoff criteria for the CFI, RMSEA, and SRMR (autoregressive RI‐CLPM: CFI = 0.969; SRMR = 0.069; RMSEA = 0.031; cross‐lagged RI‐CLPM: CFI = 0.970; SRMR = 0.071; RMSEA = 0.031), cross‐lagged paths did not appear to be necessary to achieve good model fit. Additionally, the cross‐lagged paths in the cross‐lagged RI‐CLPM were mainly small and nonsignificant (see the following sections for details). Thus, while there was no clear evidence for the superiority of either of these two models, the autoregressive RI‐CLPM was tentatively favored for the sake of avoiding overly complex models that did little to improve model fit and did not reveal any important additional parameters.

In Dataset 2 with three measurement points, the results were less clear. The AIC was similar between all models, except for a higher value of the autoregressive CLPM (autoregressive RI‐CLPM: AIC = 19,182.46; cross‐lagged RI‐CLPM: AIC = 19,181.67; autoregressive CLPM: AIC = 19,189.75; cross‐lagged CLPM: AIC = 19,181.17). However, the BIC was lowest for this model (autoregressive RI‐CLPM: BIC = 19,499.49 cross‐lagged RI‐CLPM: BIC = 19,515.83; autoregressive CLPM: BIC = 19,493.92; cross‐lagged CLPM: BIC = 19,502.48). Again, this can be explained by the fact that the BIC penalizes more complex models more heavily than the AIC and the autoregressive CLPM represented the model with the lowest complexity. Based on the fact that all models exhibited good and quite similar model fit, the simplest model, the autoregressive CLPM, might be favored for a similar reason to the autoregressive RI‐CLPM in the case with seven measurement points. However, since two out of four cross‐paths from competence beliefs on intrinsic motivation were significant in this case (see next section for details), the cross‐lagged models should not be readily rejected. Overall, no model was clearly superior to the other models in this dataset.

In summary, the autoregressive RI‐CLPM appeared to be the best fitting model in Dataset 1, and the autoregressive and cross‐lagged RI‐CLPMs appeared to be the best fitting models for Dataset 2 with seven measurement points, with the autoregressive RI‐CLPM being favored tentatively for model simplicity. In Dataset 2 with three measurement points, no model(s) were clearly superior to the others.

### 4.3 Path Coefficients

#### 4.3.1 RQ 2—Concurrent Correlations

Across all models (RI‐CLPMs, CLPMs, cross‐lagged models, autoregressive models), all concurrent correlations between competence beliefs and intrinsic motivation were positive and significant (*r* = 0.33 to 0.80, all *p* ≤ 0.004; see Table 4).

#### 4.3.2 RQ 3—Autoregressive Paths

There was a distinct difference between autoregressive paths in the RI‐CLPMs and the CLPMs. In the CLPMs, all autoregressive paths were of substantial size and statistically significant (*β* = 0.40 to 0.95, all *p* < 0.001; see Table 5). In the RI‐CLPMs, there was much more variation in the autoregressive paths (*β* = −0.03 to 0.76) and several paths were nonsignificant. There was also a clear tendency for paths to be larger in the later intervals (see below). Since in the CLPMs all autoregressive paths were positive and significant, in the following we will focus on reporting details on the more varied results in the RI‐CLPMs.

In Dataset 1, paths from competence beliefs to subsequent competence beliefs were generally positive and significant (*β* = 0.40 to 0.64, all *p* ≤ 0.024), except in the first interval of the autoregressive model (*β* = 0.27, *p* = 0.128). In the cross‐lagged model, autoregressive paths for intrinsic motivation were significant only in the last interval (*β* = 0.65, *p* = 0.011), while in the autoregressive model, all paths were significant (*β* = 0.37 to 0.59, all *p* ≤ 0.008).

In Dataset 2 with seven measurement points, the autoregressive paths for competence beliefs were positive and significant from the third interval onwards (*β* = 0.53 to 0.73, all *p* ≤ 0.001) and increased in size between almost all intervals (except between Intervals 4 and 5). In the autoregressive model, all autoregressive paths were positive and significant (*β* = 0.32 to 0.69, all *p* ≤ 0.038), and likewise increased in size between all intervals with one exception (between Intervals 1 and 2).

In Dataset 2 with three measurement points, all autoregressive paths for the second interval (4th to 7th measurement point) were positive and significant (competence beliefs: *β* = 0.55 to 0.76, all *p* ≤ 0.001; intrinsic motivation: *β* = 0.41 to 0.59, all *p* ≤ 0.007), while none of the autoregressive paths of the first interval (1st to 4th measurement point) were significant. However, it should be noted that the autoregressive path for competence beliefs in the first interval in the cross‐lagged model was descriptively large but did not reach significance due to a large standard error (*β* = 0.54, SE = 0.32, *p* = 0.095). In general, the paths' standard errors were larger in the RI‐CLPMs than in the CLPMs, possibly due to the greater complexity of the models in combination with a reduction of the variance of the involved variables after removing stable between‐person differences. This resulted in two other nonsignificant paths that should be mentioned for their size (cross‐lagged model, 2nd interval, autoregressive path of competence beliefs: *β* = 0.41, SE = 0.29, *p* = 0.148; cross‐lagged model, 2nd interval, autoregressive path of intrinsic motivation: *β* = 0.46, SE = 0.24, *p* = 0.060).

#### 4.3.3 RQ 4—Cross‐Paths

Overall, there were few significant cross‐paths in the models (see Table 6). In Dataset 1, the RI‐CLPM revealed only one significant cross‐path, namely from competence beliefs to intrinsic motivation in the first of the three intervals (*β* = 0.41, *p* = 0.035). Likewise, this was also the only significant cross‐path in the CLPM (*β* = 0.26, *p* = 0.005). In Dataset 2 with seven measurement points, the RI‐CLPM also showed only one significant cross‐path, a negative path from intrinsic motivation to competence beliefs in the third of six intervals (*β* = −0.35, *p* = 0.001). The only significant cross‐path in the CLPM was the path from competence beliefs to intrinsic motivation in the first interval (*β* = 0.18, *p* = 0.049). Notably, Dataset 2 with seven measurement points was thus the only Dataset in which the CLPM and RI‐CLPM differ in terms of their significant cross‐paths. In Dataset 2 with three measurement points, both the RI‐CLPM and the CLPM had one and the same significant cross‐path, the path from competence beliefs to intrinsic motivation in the second of the two intervals (RI‐CLPM: *β* = 0.32, *p* = 0.027; CLPM: *β* = 0.21, *p* = 0.003).

Taken together, in the RI‐CLPMs, three out of the 22 analyzed cross‐paths were statistically significant: two positive paths from competence beliefs on intrinsic motivation and one negative path from intrinsic motivation on competence beliefs. In the CLPMs, there were also three out of 22 significant cross‐paths, all positive and from competence beliefs on intrinsic motivation.

## 5 Discussion

We analyzed a long‐standing research question in educational psychology, namely the possible reciprocal relation between competence beliefs and intrinsic motivation, with a traditional (CLPM) as well as a novel method (RI‐CLPM). We also compared two different models within each of these methods: cross‐lagged models and autoregressive models, with the cross‐lagged paths fixed to zero, resulting in a total of four different model types. The results revealed that autoregressive models were generally sufficient to describe the underlying data, and RI‐CLPMs tended to outperform CLPMs but did not do so consistently across all analyses. In the following, we will first discuss differences in the model fit estimates between the four model types before turning to the path coefficients and their impact on our understanding of temporal relations between competence beliefs and intrinsic motivation.

### 5.1 RQ1—Model Comparison

The fact that the RI‐CLPMs outperformed the CLPMs in Dataset 1 and Dataset 2 with seven measurement points based on the AIC and BIC means that including random intercepts in the models improves the models' fit to the data enough to outweigh the greater complexity of the RI‐CLPMs compared with the CLPMs. Thus, this result strengthens the confidence in the notion that RI‐CLPMs are useful to analyze time‐lagged relations between constructs (Hamaker 2023; Hamaker et al. 2015; Usami et al. 2019). Beyond theoretical arguments regarding disentangling within‐ and between‐person sources of variance, they can thus also empirically improve model fit compared to CLPMs. In terms of the constructs, this means that competence beliefs and intrinsic motivation appear to possess a stable between‐person part of variance and that disentangling this stable trait portion of the constructs from intrapersonal fluctuations over time seems warranted. In Dataset 1, the autoregressive RI‐CLPM also outperformed the cross‐lagged RI‐CLPM, indicating that while the inclusion of random intercepts is warranted by a substantial improvement in fit to the data, the inclusion of cross‐lagged paths is not. In Dataset 2 with seven measurement points, the cross‐lagged and autoregressive RI‐CLPMs performed similarly. Thus, whether cross‐lagged paths should be included in this case cannot unequivocally be answered based on information criteria alone; although the simpler autoregressive RI‐CLPM was tentatively favored for model parsimony. The results of the model comparisons were least clear in Dataset 2 with three measurement points, where no model(s) clearly outperformed the others.

Given that almost all models performed well in the present study, we will discuss and compare the results of the different models in the following sections. In particular, we will focus on different interpretations between path coefficients from RI‐CLPMs and CLPMs.

### 5.2 RQ2—Concurrent Correlations

In the present study, all concurrent correlations between competence beliefs and intrinsic motivation were positive and significant. These correlations ranged from *r* = 0.33 to *r* = 0.80 with an average of *M*_r_ = 0.61. This result is of comparable size to the concurrent correlations observed in prior studies (e.g., *r* = 0.43 to *r* = 0.58 (Arens et al. 2019); *r* = 0.48 to *r* = 0.59 (Niemivirta et al. 2023); *r* = 0.29 to *r* = 0.83 (Spinath and Steinmayr 2008); *r* = 0.36 to *r* = 0.51 (Steinmayr et al. 2018); *r* = 0.73 (Sáinz and Upadyaya 2023); *r* = 0.67 to *r* = 0.70 (Vinni‐Laakso et al. 2019)). The correlations between the random intercepts in the RI‐CLPMs were somewhat larger (ranging from *r* = 0.63 to *r* = 0.89 with an average of *M*_r_ = 0.76).

Spinath and Steinmayr (2008) argued that the reason for high concurrent correlations between competence beliefs and intrinsic motivation might be that certain experiences, such as unfavorable feedback, affect both constructs at the same time. The fact that we observed substantial and consistent associations between competence beliefs and intrinsic motivation not only in the CLPMs but also in the RI‐CLPMs is in line with this reasoning. From a between‐person perspective, unobserved time‐invariant covariates (e.g., gender, migration background, socioeconomic status (SES), and others) might be an additional or alternative explanation to the one given by Spinath and Steinmayr (2008). However, path coefficients in RI‐CLPMs are more robust against the omission of time‐invariant covariates (Hamaker 2023; Hamaker et al. 2015; Marsh et al. 2022). Thus, the fact that consistent and significant concurrent correlations were observed in RI‐CLPMs as well, strengthens the confidence that these correlations are not (only) the result of time‐invariant covariates (e.g., gender, migration background, or SES; assuming that the covariates as well as their influences on the variables under consideration are indeed time‐invariant factors). If they are instead time‐varying (e.g., because effects of gender stereotypes in mathematics become more pronounced with age), the time‐varying portion of their influence can affect within‐person associations. Thus, alternative explanations for effects of certain experiences as discussed by Spinath and Steinmayr (2008) cannot be ruled out entirely. Additionally, the random intercepts in the RI‐CLPMs also exhibit positive and significant correlations. Students with a high stable trait‐like level competence beliefs also tend to have a high stable trait‐like level of intrinsic motivation. Thus, competence beliefs and intrinsic motivation appear to be positively related both on the level of the temporally stable traits and on the level of temporally variable states.

### 5.3 RQ3—Autoregressive Relations

Autoregressive paths reflect temporal between‐person stability in CLPMs. Thus, since all autoregressive paths in the CLPMs are positive, significant, and of substantial size (*β* = 0.40 to 0.95) in the CLPMs, both competence beliefs and intrinsic motivation appear to be fairly stable across time between persons. In RI‐CLPMs, however, autoregressive paths reflect whether deviations from a person's expected score in a certain construct can be predicted by prior deviations from the expected score in the same construct (Hamaker et al. 2015; Marsh et al. 2022; Usami et al. 2019). In other words, they reflect the extent to which these deviations are carried over to the next time point (hence the term “carry‐over effect”; Hamaker et al. 2015, 104). Our results indicate that carry‐over effects mostly increase with grades. Autoregressive paths in the RI‐CLPMs are generally small to moderate in size (mostly between *β* = 0.10 and *β* = 0.49) and sometimes nonsignificant in the early intervals, but consistently at or above *β* = 0.50 and significant in the later intervals (with the only exception being the autoregressive path for intrinsic motivation in the second interval in Dataset 2 with three measurement points with *β* = 0.41, *p* = 0.007; for details, see Table 5). A possible explanation is that because with increasing age students improve in their cognitive ability to incorporate information (e.g., verbal feedback, grades, observed competence of peers) into the formation of more stable competence beliefs (Wigfield and Eccles, 2002), such information could have a longer lasting impact on older than younger students. The competence beliefs of younger students might instead depend more strongly on recent experiences instead of an aggregate of the information obtained over a prolonged time period. Regarding intrinsic motivation, students might form more stable interests in certain types of tasks and academic domains they enjoy through an increasing number of positive or negative experiences with the respective tasks and domains across their school career. In other words, making specific experiences with a subject (e.g., receiving a good grade in math, not being able to solve a homework assignment in math, having fun solving a math problem, etc.) can affect students competence beliefs and intrinsic motivation. If they affect them for a long enough time (i.e., until the next measurement point) this could explain the observed autoregressive paths and thus the carry‐over effects in the RI‐CLPMs.

There are also other possible explanations for the significant autoregressive paths in the RI‐CLPMs. Students' motivation and self‐concepts typically decline throughout the school career (e.g., Engler and Westphal 2024; Freiberger and Spinath 2014; Gaspard et al. 2020; Gnambs and Hanfstingl 2016; Scherrer and Preckel 2019). Stage–environment–fit theory suggests that this decline might be the result of increasing discrepancies between students' needs (e.g., for autonomy) and the school environment, which often does not allow students to fulfill these needs (Eccles et al. 1993). Thus, we cannot unequivocally answer what caused the significant carry‐over effects, particularly in older students. Older students might be affected by specific events such as grades and feedback for a longer time than younger students or the competence beliefs and intrinsic motivation of older students might continuously decline because of a mismatch between their psychological needs and the school environment, both of which could cause observed carry‐over effects.

### 5.4 RQ4—Lagged Relations

Considering only the cross‐lagged models, we analyzed an overall of 22 cross‐paths between competence beliefs and intrinsic motivation once with RI‐CLPMs and once with CLPMs. Across all models only three of the 22 cross‐paths were significant (although not always the same cross‐paths). In Dataset 1 and Dataset 2 with three measurement points, the same cross‐paths were significant between the RI‐CLPMs and CLPMs while in Dataset 2 with seven measurement points, the results did differ. In Dataset 2 with seven measurement points, one of the six cross‐paths from competence beliefs to intrinsic motivation was positive and significant in the CLPM (first of the six intervals). In the RI‐CLPM, there was one negative and significant path from intrinsic motivation to competence belief (third of six intervals). However, the positive and significant cross‐path from competence beliefs to intrinsic motivation in the CLPM only minimally surpassed the 5% alpha threshold (*β* = 0.18, *p* = 0.049). Additionally, the negative cross‐path from intrinsic motivation to competence beliefs is the only significant cross‐path from intrinsic motivation to competence beliefs out of the 22 analyzed cross‐paths. Thus, there is not enough evidence that the differences between these models constitute more than random noise due to the relatively large number of estimated models and parameters.

Overall, with only three out of 22 cross‐paths being significant in either model type, there is little evidence that intrinsic motivation can be predicted from prior competence beliefs or vice versa. However, three additional points should be considered.

First, while most cross‐paths were nonsignificant, there were nonetheless six significant ones that warrant consideration, especially since five of these cross‐paths showed that prior competence beliefs predict higher subsequent intrinsic motivation as assumed in expectancy‐value theory (Eccles and Wigfield 2023; Wigfield and Eccles 2020), the model of effectance motivation by Harter (1981), and self‐determination theory (Ryan and Deci 2019, 2020). This was the case in the first interval in Dataset 1 (both RI‐CLPM and CLPM), the first interval in Dataset 2 with seven measurement points (only CLPM) and the second interval in Dataset 2 with three measurement points (both RI‐CLPM and CLPM). However, most of these cross‐paths stem from models that were outperformed by other models which either did not include cross‐paths at all (the autoregressive RI‐CLPM in Dataset 1 and Dataset 2 with seven measurement points) or had no significant cross‐paths (cross‐lagged RI‐CLPM in Dataset 2 with seven measurement points). Additionally, the fact that Dataset 2 revealed entirely different cross‐paths when seven measurement points were used (*t*_1_ to *t*_2_) and when three measurement points were used (*t*_4_ to *t*_7_) shows that these cross‐paths are highly inconsistent. Thus, while these cross‐paths might suggest that there is some predictive power of competence beliefs for intrinsic motivation as early as second grade, they should be interpreted with caution.

Second, cross‐paths in RI‐CLPMs and cross‐paths in CLPMs require different interpretations. The lack of consistent cross‐paths in the CLPMs indicates that students with high competence beliefs relative to their peers at one point in time do not tend to have particularly high or low intrinsic motivation relative to their peers at the next point in time or vice versa. The lack of consistent cross‐paths in the RI‐CLPMs indicates that students with high competence beliefs at one point in time relative to their usual competence beliefs do not tend to have particularly high or low intrinsic motivation at the next point in time relative to their usual intrinsic motivation or vice versa. Thus, by employing both CLPMs and RI‐CLPMs we could show that neither a combination of within‐ and between‐person variance in one construct (what Marsh et al. 2022 calls “Undecomposed Between‐Person (CLPM) Effects”, p.2727) nor within‐person variance in one construct (what Marsh et al. 2022 calls “Within‐Person (RI‐CLPM) … Effects”, p.2727) predicts the respective other construct consistently.

Third, the standard errors in the RI‐CLPM are generally larger than in the CLPMs and also vary substantially between the paths. This resulted in some cross‐path being relatively large (up to *β* = −0.37, see Table 6) and nonsignificant in the RI‐CLPMs. This was likely the case since RI‐CLPMs are more complex than CLPMs and thus, the sample sizes of the two datasets might have been low for this type of model. Mulder (2023) reports results from a simulation study indicating necessary sample sizes of 1000 or more to detect cross‐lagged effects of 0.10 with 80% power in RI‐CLPMs, which the two datasets did not meet. However, the fact that the CLPMs with smaller standard errors produced similar results for the cross‐paths, supports the interpretation that there is indeed little evidence that prior competence beliefs predict subsequent intrinsic motivation or vice versa.

Given these results, what are possible explanations for the apparent absence of consistent lagged relations between competence beliefs and intrinsic motivation? First, one reason might be the restricted variance in competence beliefs and intrinsic motivation in young students. Elementary school students typically exhibit high levels of competence beliefs and intrinsic motivation (e.g., Niemivirta et al. 2023; Spinath and Steinmayr 2008; Weidinger et al. 2018). Indeed, students in both samples analyzed in the present study had high intrinsic motivation and competence beliefs at all measurement points (competence beliefs: *M* = 3.78 to 4.05; intrinsic motivation: *M* = 3.69 to 4.00; scales from 1 to 5). In particular, there were few students with low values in their competence beliefs and to a lesser extent, few students with low values in intrinsic motivation. Thus, the restricted variance and lack of students with low values in the constructs might have negatively impacted the cross‐lagged paths. Notably, because RI‐CLPMs are concerned with within‐person effects, this argument pertains to restricted variance within students, not between students, as well. If an individual student has high competence beliefs in mathematics at all measurement points, then there is little within‐person variance in their competence beliefs and thus, their competence beliefs might not explain their subsequent intrinsic motivation.

Second, following the argument by Spinath and Steinmayr (2008), experiences of intrinsic motivation might be possible across a broad spectrum of competence beliefs as long as task difficulty is suited for the students' level of competence and competence beliefs. For example, students who do not consider their own mathematical competencies as high might enjoy mathematical tasks if their teachers provide individualized feedback and tasks that are easy enough for students to solve but challenging enough to allow for feelings of earned success and efficacy (see Usher and Pajares 2008). If this is the case, the absolute level of competence beliefs might have little impact on students' intrinsic motivation. Students who consider themselves highly competent might still experience low motivation if they feel unchallenged in their classes and students who consider themselves less competent might experience high motivation if the tasks provided by the teachers are challenging but solvable for them. Thus, the relation between competence beliefs, actual competence, and task difficulty might be more important for intrinsic motivation than the absolute level of competence beliefs, possibly explaining the low number of significant cross‐paths in the present study.

Lastly, feelings of competence are only one of humans' innate psychological needs alongside autonomy, social relatedness (Ryan and Deci 2019, 2020), and possibly others (e.g., Fernández‐Espínola et al. 2020; González‐Cutre et al. 2016, 2020). Autonomy, for example, becomes more important with age and autonomy support from teachers can counteract the common decrease in motivation including in elementary school (e.g., Baten et al. 2020; Fu et al. 2023; Miwa and Toyama 2021). Thus, predicting intrinsic motivation by competence beliefs alone might be too narrow of a view.

### 5.5 Limitations and Future Directions

The present study is the first to employ RI‐CLPMs in the analysis of lagged relations between competence beliefs and intrinsic motivation. We analyzed data from German elementary school students in second to third (Dataset 1) and second to fourth (Dataset 2) grade in mathematics. Therefore, replications in future studies, particularly in different domains (e.g., reading) and populations (e.g., secondary school students, students from different countries) are needed to validate and generalize the results.

Furthermore, the choice of the specific time points of measurement and the intervals between them might have a pronounced impact on the results, especially if carry‐over effects are small. If high (or low) competence beliefs are not carried over across time to a large extent, the measured competence beliefs at a certain point in time (e.g., *t*_1_) might not be closely related to the usual competence beliefs within the following interval (*t*_1_ to *t*_2_) and, thus, have little impact on intrinsic motivation at the next time point (*t*_2_). This could be the case if students' deviations from their usual trait‐like competence beliefs are strongly influenced by recent information (e.g., feedback from teachers, peers, or parents) or their mood and to a lesser extent by the collective information over a larger time period. This problem might be more pronounced in RI‐CLPMs compared to CLPMs because in CLPMs, the latent scores at each time point are composed of both, the person's usual trait‐like level in the respective construct and the deviation from that trait‐like level (“undecomposed between person effects”; Marsh et al. 2022; 2704), while in an RI‐CLPM, they are composed only of the deviation from the trait‐like level. In other words, since the stable portion of the respective construct is controlled for in RI‐CLPMs, the individual scores more strongly depend on the specific measurement time point. This potential problem could be mitigated through diary studies in which students report their competence beliefs and intrinsic motivation repeatedly within certain intervals. Then, an aggregate of responses within a certain time interval could be used to predict an aggregate of responses within the following time interval. Such a procedure seems especially feasible since competence beliefs and intrinsic motivation can both be assessed reliably with a small number of items (often 3 or 4 and sometimes only 2; e.g., Arens and Niepel 2023; Spinath and Steinmayr 2008; Weidinger et al. 2018).

The specific time points of the measurement could have also affected the results due to seasonal changes. It is possible that students were more motivated and had a more favorable perception of their own competencies at certain times in a school year (e.g., right after a break period) than at other times. This would constitute a time‐varying factor that affects intrinsic motivation and competence beliefs and could have therefore affected the results in both the RI‐CLPMs and the CLPMs. Notably, in the analysis of Dataset 2 with three measurement points, we used three measurement points that were 1 year apart (all in July, see Table 1). Thus, seasonal changes cannot explain the results in these analyses. Their similarity to the results for Dataset 1 and Dataset 2 with all seven measurement points strengthens the confidence in the robustness of the conclusions drawn.

Another limitation is the fact that we could not control for the nested data structure in Dataset 2. However, the proportion of variance resulting from the clustered data was small.

The sample sizes of the two datasets might have been too small to detect small cross‐lagged effects. This might also have been the reason for some large standard errors in the RI‐CLPMs. Notably, Mulder (2023) reports a negative impact of missing and nonnormal data on statistical power. Thus, especially given the lack of students with low scores in intrinsic motivation and/or competence beliefs, the sample sizes of *N* = 664 and *N* = 542 might have been too small to detect cross‐lagged effects of small size. Since the present study is a re‐analysis of existing datasets previously analyzed with traditional CLPMs, sample sizes were not planned for the application of RI‐CLPMs. Researchers assessing data for the application of RI‐CLPMs should take these considerations regarding statistical power into account. In particular, when intrinsic motivation and/or competence beliefs are assessed in young students, larger sample sizes might be needed because young students often exhibit high values in these constructs, resulting in skewed distributions of the constructs.

Lastly, the results are of course influenced by the choice of datasets to be analyzed. This is especially relevant in the present study since a dataset was chosen for analysis that did not reveal many significant cross‐paths between competence beliefs and intrinsic motivation in a prior analysis. Because of this, we validated the results of the first dataset with a second dataset which was not previously analyzed for this research question. Still, the overall results and interpretation of the present study might have differed, had we selected a dataset in which cross‐paths between the constructs were observed using CLPMs (e.g., Arens et al. 2019). Thus, future studies are needed to further validate the results of the present study with datasets in which such cross‐paths were found. This in conjunction with larger sample sizes and possibly combining multiple datasets would be helpful to investigate the robustness and generalizability of the present results.

### 5.6 Conclusion

Overall, our results indicate very little evidence for cross‐lagged associations between elementary school students' competence beliefs and intrinsic motivation in mathematics, neither on a within‐person level nor on an undecomposed between‐person level. These results consequently echo previous studies that found similar results for undecomposed between‐person associations in elementary school students (e.g., Spinath and Steinmayr 2008; Vinni‐Laakso et al. 2019; Weidinger et al. 2018). Additionally, the results do not differ much between RI‐CLPMs and CLPMs, neither in terms of model fit nor in terms of the path coefficients. While the RI‐CLPMs did outperform the CLPMs in two out of three cases (Dataset 1 and Dataset 2 with seven measurement points), most models achieved good and similar model fits, and both models yielded similar substantive conclusions drawn. Thus, the choice of model type might not have a large impact in the case of analyzing time‐shifted relations between competence beliefs and intrinsic motivation. We recommend that researchers carefully evaluate which processes they deem important for theorized relations between competence beliefs and intrinsic motivation. Specifically, if there is a theoretical reason to assume that within‐person variance in one variable is associated with within‐person variance in the other variable (or if researchers suspect potential unobserved covariates that could impact the results), RI‐CLPMs are likely preferable. Most importantly, however, researchers should be aware that results of the two model types need to be interpreted differently.

## Conflicts of Interest

The authors declare no conflicts of interest.

## Supporting information


**Data S1:** cdev70028‐sup‐0001‐supinfo.docx.

## Data Availability

The data analyzed in this article are not publically available.
