# Supplementary material for: Re‐Examination of the Reciprocal Relation Between Intrinsic Motivation and Competence Beliefs in Math Using the Random‐Intercept Cross‐Lagged Panel Framework
Source: Child Dev. 2025 Sep 4;96(6):2201–20. doi: 10.1111/cdev.70028 (PMC12598449; doi:10.1111/cdev.70028)
Supplement: Supplementary file 1 — Data S1: cdev70028‐sup‐0001‐supinfo.docx. [file CDEV-96-2201-s001.docx]

**Online Supplemental Material**

**Re-Examination of the Reciprocal Relation Between Intrinsic Motivation and Competence Beliefs in Math Using the Random-Intercept Cross-Lagged Panel Framework.**

**By blinded for peer review, submitted, *Child Development***

**Overview**

Supplement 1. Results of the Analysis of Measurement Invariance

Supplement 2. Histograms of the Individual Items

Supplement 3. Paths from Gender on Competence Beliefs and Intrinsic Motivation

**OSM 1 – Results of the Analysis of Measurement Invariance**

**Table S1**

Results for the multi-group CFAs on four levels of measurement invariance.

| Model Dataset 1 (Spinath & Steinmayr, 2008) | χ² (df) | CFI | RMSEA | SRMR | Δχ2 (Δdf) | ΔCFI | ΔRMSEA | ΔSRMR | Decision |
| --- | --- | --- | --- | --- | --- | --- | --- | --- | --- |
| Intrinsic Motivation |  |  |  |  |  |  |  |  |  |
| Configural invariance | 44.06 (31) | .993 | .025 | .021 | - | - | - | - | Accepted |
| Metric invariance | 49.05 (37) | .994 | .022 | .025 | 4.99 (6) | .001 | -.003 | .004 | Accepted |
| Scalar invariance | 63.37 (43)* | .990 | .027 | .028 | 14.32 (6) | -.004 | .005 | .003 | Accepted |
| Residual Invariance | 75.14 (52)* | .988 | .026 | .033 | 11.77 (9) | -.002 | -.001 | .005 | Accepted |
| Competence beliefs |  |  |  |  |  |  |  |  |  |
| Configural invariance | 47.40 (31) | .994 | .028 | .025 | - | - | - | - | Accepted |
| Metric invariance | 63.35 (37)* | .991 | .033 | .042 | 15.95 (6) | -.003 | .005 | .017 | Accepted |
| Scalar invariance | 65.74 (43)* | .992 | .028 | .043 | 2.39 (6) | .001 | -.005 | .001 | Accepted |
| Residual Invariance | 99.76 (52)* | .983 | .037 | .051 | 34.02 (9) | -.009 | .009 | .008 | Accepted |
| Model Dataset 2 (Weidinger et al., 2018) | χ² (df) | CFI | RMSEA | SRMR | Δχ2 (Δdf) | ΔCFI | ΔRMSEA | ΔSRMR | Decision |
| Intrinsic Motivation |  |  |  |  |  |  |  |  |  |
| Configural invariance | 147.74 (105)** | .992 | .027 | .033 | - | - | - | - | Accepted |
| Metric invariance | 161.31 (117)** | .991 | .026 | .035 | 13.57 (12) | -.001 | -.001 | .002 | Accepted |
| Scalar invariance | 187.31 (129)*** | .988 | .029 | .035 | 26.00 (12) | -.003 | .003 | .000 | Accepted |
| Residual Invariance | 298.62 (147)*** | .970 | .044 | .047 | 111.31 (18) | -.018 | .015 | .012 | Rejected |
| Competence beliefs |  |  |  |  |  |  |  |  |  |
| Configural invariance | 103.37 (105) | 1.000 | .000 | .020 | - | - | - | - | Accepted |
| Metric invariance | 114.75 (117) | 1.000 | .000 | .031 | 11.38 (12) | .000 | .000 | .011 | Accepted |
| Scalar invariance | 158.69 (129)* | .994 | .021 | .031 | 43.94 (12) | -.006 | .021 | .000 | Rejected |
| Residual Invariance | 279.30 (147)*** | .975 | .041 | .046 | 120.61 (18) | -.019 | .020 | .015 | Rejected |

**OSM 2 – Histograms of the Individual Items**

**Figure S2**

Histograms of the individual competence beliefs and intrinsic motivation items in Dataset 1 (Spinath & Steinmayr, 2008) and Dataset 2 (Weidinger et al., 2018)


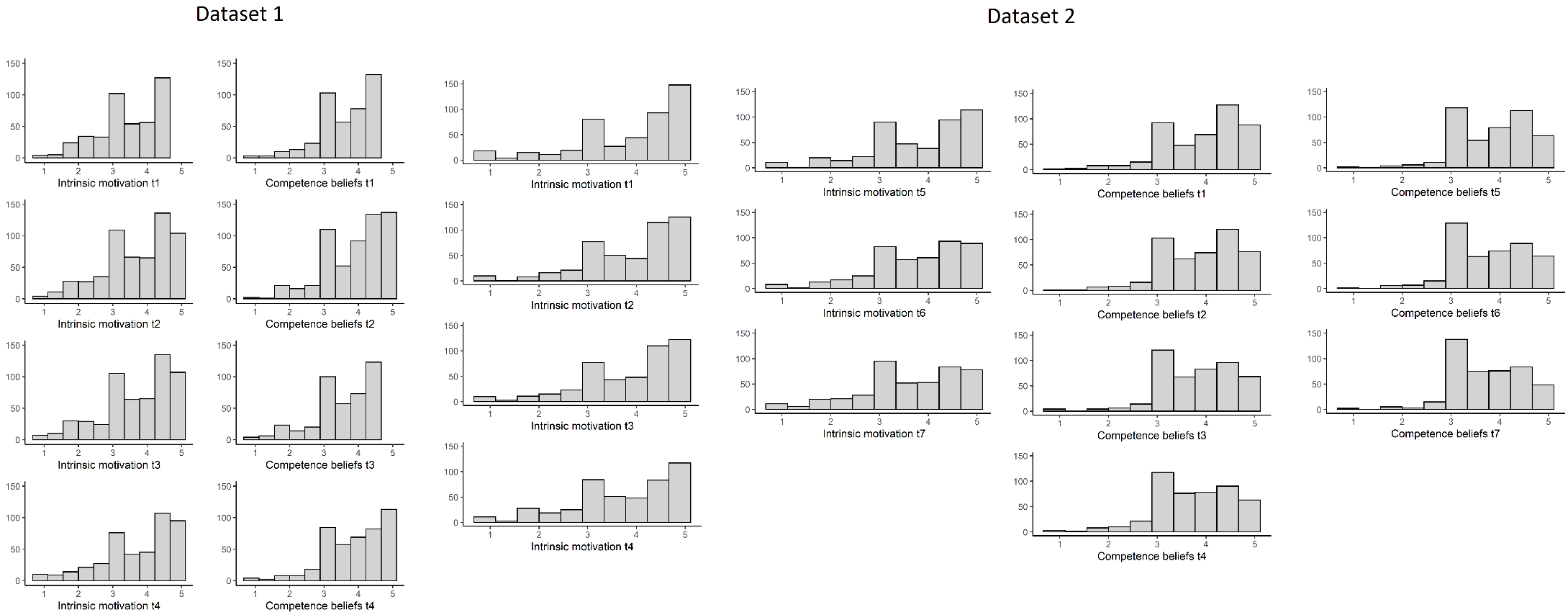


**OSM 3 – Paths from Gender on Competence Beliefs and Intrinsic Motivation**

All paths from gender on competence beliefs and intrinsic motivation are reported in Table S3. Across all models (RI-CLPMs, CLPMs, cross-lagged models, autoregressive models), there are several significant but mostly small paths from gender to competence beliefs and intrinsic motivation (range: β = -.34 to .06). Almost all paths are negative, indicating lower values for girls than for boys after controlling for the other variables. Descriptively, there is a trend for paths to become smaller in later intervals in all Datasets (see Table S3). In Dataset 1, all paths from gender to competence beliefs are statistically significant in all models (β = -.34 to -.04, all *p* ≤ .025), while only nine of 16 paths to intrinsic motivation are significant (β = -.21 to -.02, *p* = .001 to .283). In Dataset 2 with seven measurement points, the results for paths to competence beliefs are not as consistent as in Dataset 1. Here, 16 of 28 paths from gender to competence beliefs are statistically significant, with coefficients of all paths (significant or nonsignificant) ranging from β = -.26 to .06 (*p* = .001 to .852). For intrinsic motivation, 21 of 28 paths from gender are significant with coefficients of all paths ranging from β = -.21 to .02 (*p* = .001 to .596). Lastly, in Dataset 2 with three measurement points, 10 out of 12 paths from gender to competence beliefs are significant (β = -.25 to -.06, *p* = .001 to .164). For intrinsic motivation, 11 out of 12 paths are significant (β = -.17 to -.05, *p* = .001 to .080). Thus, overall, there is evidence in all datasets that being female is associated with lower competence beliefs and lower intrinsic motivation in mathematics.

**Table S3**

Paths from gender on competence beliefs (CB) and intrinsic motivation (IV) in all models

|  | RI-CLPM | | | | | | CLPM | | | | | |
| --- | --- | --- | --- | --- | --- | --- | --- | --- | --- | --- | --- | --- |
|  | Cross-lagged model | | | Autoregressive model | | | Cross-lagged model | | | Autoregressive model | | |
| Dataset 1 | β | *SE* | *p* | β | *SE* | *p* | β | *SE* | *p* | β | *SE* | *p* |
| *t*_S1_ CB | -.32 | .05 | < .001 | -.34 | .06 | < .001 | -.22 | .03 | < .001 | -.22 | .03 | < .001 |
| *t*_S2_ CB | -.21 | .06 | < .001 | -.28 | .09 | .001 | -.08 | .02 | < .001 | -.08 | .02 | < .001 |
| *t*_S3_ CB | -.11 | .05 | .025 | -.14 | .04 | .001 | -.04 | .01 | .002 | -.04 | .01 | < .001 |
| *t*_S4_ CB | -.20 | .05 | < .001 | -.20 | .04 | < .001 | -.07 | .02 | < .001 | -.07 | .01 | < .001 |
| *t*_S1_ IV | -.21 | .08 | .011 | -.20 | .08 | .010 | -.12 | .05 | .008 | -.13 | .05 | .007 |
| *t*_S2_ IV | -.09 | .07 | .183 | -.16 | .06 | .009 | -.02 | .02 | .133 | -.05 | .01 | < .001 |
| *t*_S3_ IV | -.10 | .08 | .219 | -.14 | .06 | .025 | -.02 | .02 | .283 | -.03 | .02 | .102 |
| *t*_S4_ IV | -.09 | .03 | .006 | -.08 | .04 | .040 | -.02 | .01 | .105 | -.02 | .01 | .050 |
|  | Cross-lagged model | | | Autoregressive model | | | Cross-lagged model | | | Autoregressive model | | |
| Dataset 2, 7 time points | β | *SE* | *p* | β | *SE* | *p* | β | *SE* | *p* | β | *SE* | *p* |
| *t*_W1_ CB | -.26 | .06 | < .001 | -.26 | .06 | < .001 | -.19 | .05 | < .001 | -.19 | .05 | < .001 |
| *t*_W2_ CB | -.24 | .07 | .001 | -.23 | .07 | .002 | -.06 | .04 | .173 | -.07 | .04 | .098 |
| *t*_W3_ CB | -.23 | .09 | .015 | -.23 | .09 | .010 | -.04 | .04 | .352 | -.04 | .04 | .339 |
| *t*_W4_ CB | -.29 | .09 | .002 | -.25 | .07 | < .001 | -.09 | .03 | .007 | -.10 | .03 | .003 |
| *t*_W5_ CB | -.15 | .08 | .075 | -.12 | .07 | .094 | -.01 | .03 | .852 | -.01 | .04 | .841 |
| *t*_W6_ CB | -.02 | .07 | .810 | -.02 | .06 | .748 | .06 | .03 | .084 | .05 | .03 | .146 |
| *t*_W7_ CB | -.23 | .05 | < .001 | -.22 | .05 | < .001 | -.12 | .03 | < .001 | -.12 | .03 | < .001 |
| *t*_W1_ IV | -.12 | .05 | .009 | -.13 | .05 | .007 | -.10 | .04 | .005 | -.10 | .04 | .005 |
| *t*_W2_ IV | -.05 | .06 | .377 | -.11 | .05 | .041 | -.01 | .02 | .596 | -.03 | .02 | .086 |
| *t*_W3_ IV | -.22 | .07 | .003 | -.14 | .06 | .030 | -.05 | .02 | .005 | -.04 | .02 | .012 |
| *t*_W4_ IV | -.16 | .07 | .034 | -.13 | .04 | .002 | -.05 | .01 | .001 | -.06 | .01 | < .001 |
| *t*_W5_ IV | -.10 | .06 | .103 | -.03 | .03 | .344 | .01 | .01 | .304 | .01 | .01 | .305 |
| *t*_W6_ IV | -.13 | .06 | .022 | -.12 | .04 | .001 | -.04 | .02 | .013 | -.05 | .01 | < .001 |
| *t*_W7_ IV | -.15 | .04 | < .001 | -.12 | .03 | < .001 | -.06 | .01 | < .001 | -.06 | .01 | < .001 |
|  | Cross-lagged model | | | Autoregressive model | | | Cross-lagged model | | | Autoregressive model | | |
| Dataset 2, 3 time points | β | *SE* | *p* | β | *SE* | *p* | β | *SE* | *p* | β | *SE* | *p* |
| *t*_W1_ CB | -.20 | .07 | .002 | -.25 | .07 | < .001 | -.18 | .05 | < .001 | -.18 | .05 | < .001 |
| *t*_W4_ CB | -.18 | .08 | .025 | -.32 | .11 | .003 | -.15 | .04 | .001 | -.16 | .04 | < .001 |
| *t*_W7_ CB | -.08 | .06 | .164 | -.16 | .06 | .007 | -.06 | .03 | .077 | -.07 | .04 | .034 |
| *t*_W1_ IV | -.14 | .05 | .005 | -.14 | .05 | .005 | -.11 | .04 | .004 | -.11 | .04 | .004 |
| *t*_W4_ IV | -.13 | .06 | .044 | -.17 | .07 | .010 | -.09 | .03 | < .001 | -.10 | .02 | < .001 |
| *t*_W7_ IV | -.08 | .05 | .080 | -.14 | .06 | .021 | -.05 | .02 | .007 | -.08 | .02 | < .001 |
